# Development of an anti-bear podoplanin monoclonal antibody PMab-247 for immunohistochemical analysis

**DOI:** 10.1016/j.bbrep.2019.100644

**Published:** 2019-04-28

**Authors:** Yoshikazu Furusawa, Junko Takei, Yusuke Sayama, Shinji Yamada, Mika K. Kaneko, Yukinari Kato

**Affiliations:** a Department of Antibody Drug Development, Tohoku University Graduate School of Medicine, 2-1 Seiryo-machi, Aoba-ku, Sendai, Miyagi, 980-8575, Japan; b New Industry Creation Hatchery Center, Tohoku University, 2-1 Seiryo-machi, Aoba-ku, Sendai, Miyagi, 980-8575, Japan

**Keywords:** Bear podoplanin, PDPN, Immunohistochemistry, PMab-247, BAP, bovine aggrus/podoplanin, DAB, 3,3′-diaminobenzidine tetrahydrochloride, ELISA, enzyme-linked immunosorbent assay, PBS, phosphate-buffered saline, PDPN, podoplanin, mAb, monoclonal antibody

## Abstract

Sensitive and specific monoclonal antibodies (mAbs) targeting podoplanin (PDPN) are needed for immunohistochemical analyses using formalin-fixed paraffin-embedded tissues because PDPN is known as a lymphatic endothelial cell maker in pathology. Recently, we established *anti*-PDPN mAbs against many species, such as human, mouse, rat, rabbit, dog, cat, bovine, pig, horse, goat, tiger, alpaca, and Tasmanian devil. However, anti-bear PDPN (bPDPN) has not been established yet. In this study, we immunized mice with bPDPN-overexpressing Chinese hamster ovary (CHO)-K1 (CHO/bPDPN) cells, and screened mAbs against bPDPN using flow cytometry. One of the mAbs, PMab-247 (IgG_1_, kappa), specifically detected CHO/bPDPN cells by flow cytometry and immunohistochemistry. Our findings suggest the potential usefulness of PMab-247 for the functional analyses of bPDPN.

## Highlights

• PDPN is known as a specific lymphatic endothelial cell (LEC) marker.
• Sensitive and specific PMab-247 mAb against bear PDPN was produced.
• PMab-247 strongly reacted with bear PDPN in flow cytometry.
• PMab-247 is useful for IHC using paraffin-embedded cell sections.

## 1 Introduction

A type I transmembrane sialo-glycoprotein, podoplanin (PDPN), is expressed in many cell types, such as renal podocytes, pulmonary type I alveolar cells, and lymphatic endothelial cells of every organ [1]. PDPN has been reported to distinguish lymphatic endothelial cells from vascular endothelial cells in pathophysiological studies [2]. C-type lectin-like receptor-2 (CLEC-2) was previously reported as an endogenous receptor of PDPN in our studies [3,4]. The PDPN-CLEC-2 interaction facilitates the separation of embryonic blood and lymphatic vessels [5]. Human PDPN (hPDPN) is expressed in many malignant tumors, including brain tumors [6] and mesotheliomas [7], and is associated with malignant progression and cancer metastasis [8].

We have developed monoclonal antibodies (mAbs) against human [6], mouse [9], rat [10], rabbit [11], bovine [12], dog [13], cat [14], pig [15], horse [16], goat [17], tiger [18], alpaca [19], whale [20], and Tasmanian devil [21] PDPNs. In this study, we immunized mice with CHO/bear PDPN (bPDPN) cells and established mAbs against bPDPN.

## 2 Materials and methods

### 2.1 Cell lines

CHO-K1 and P3X63Ag8U.1 (P3U1) cells were obtained from the American Type Culture Collection (ATCC; Manassas, VA, USA). Synthesized DNA (Eurofins Genomics KK, Tokyo, Japan) encoding bPDPN (accession No.: XM_008694703.1) plus an N-terminal BAP tag, which are recognized by an *anti*-BAP tag mAb (PMab-44), was subcloned into a pCAG-Neo vector (FUJIFILM Wako Pure Chemical Corporation, Osaka, Japan). Plasmids were transfected using Lipofectamine LTX with Plus Reagent (Thermo Fisher Scientific Inc., Waltham, MA, USA). Stable transfectants (CHO/bPDPN) were selected by limiting dilution and cultivation in a medium containing 0.5 mg/mL of G418 (Nacalai Tesque, Inc., Kyoto, Japan).

The P3U1, CHO-K1, CHO/bPDPN, CHO/hPDPN [22], CHO/mouse PDPN (mPDPN) [22], CHO/rat PDPN (rPDPN) [10], CHO/rabbit PDPN (rabPDPN) [11], CHO/dog PDPN (dPDPN) [13], CHO/bovine PDPN (bovPDPN) [12], CHO/cat PDPN (cPDPN) [14], CHO/pig PDPN (pPDPN) [15], CHO/horse PDPN (horPDPN) [16], CHO/tiger PDPN (tigPDPN) [18], CHO/alpaca PDPN (aPDPN) [19], CHO/Tasmanian devil PDPN (tasPDPN) [21], CHO/goat PDPN (gPDPN) [17], CHO/sheep PDPN (sPDPN) [23], and CHO/whale PDPN (wPDPN) [20] were cultured in a Roswell Park Memorial Institute (RPMI) 1640 medium (Nacalai Tesque, Inc.), supplemented with 10% of heat-inactivated fetal bovine serum (FBS; Thermo Fisher Scientific Inc.), 100 units/mL of penicillin, 100 μg/mL of streptomycin, and 25 μg/mL of amphotericin B (Nacalai Tesque, Inc.). The cells were grown in an incubator at 37 °C with humidity and 5% CO_2_ and 95% air atmosphere.

### 2.2 Animals

Female BALB/c mice (6 weeks of age) were purchased from CLEA Japan (Tokyo, Japan). The animals were housed under specific pathogen-free conditions. The Animal Care and Use Committee of Tohoku University approved all animal experiments.

### 2.3 Hybridoma production

We employed a Cell-Based Immunization and Screening (CBIS) method [14,[24], [25], [26]] to develop mAbs against bPDPN. One BALB/c mouse was immunized with CHO/bPDPN cells (1 × 10^8^) intraperitoneally (i.p.) together with the Imject Alum (Thermo Fisher Scientific Inc.). The procedure included three additional immunizations of CHO/bPDPN cells (1 × 10^8^) together with the Imject Alum, followed by a final booster injection of CHO/bPDPN cells (1 × 10^8^) together with the Imject Alum 2 days prior to the harvest of spleen cells. Furthermore, one BALB/c mouse was immunized with CHO/bPDPN cells (1 × 10^8^) intraperitoneally (i.p.) together with chitosan (Koyo Chemical Co., Ltd., Osaka, Japan). The procedure included three additional immunizations of CHO/bPDPN cells (1 × 10^8^) together with chitosan, followed by a final booster injection of CHO/bPDPN cells (1 × 10^8^) together with chitosan 2 days prior to the harvest of spleen cells. Subsequently, these spleen cells were fused with P3U1 cells using PEG1500 (Roche Diagnostics, Indianapolis, IN, USA), and the hybridomas were grown in an RPMI medium supplemented with hypoxanthine, aminopterin, and thymidine (HAT) for selection (Thermo Fisher Scientific Inc.). The culture supernatants were screened by flow cytometry.

### 2.4 Flow cytometry

The cells were harvested following a brief exposure to 0.25% trypsin and 1 mM ethylenediaminetetraacetic acid (EDTA; Nacalai Tesque, Inc.). The cells were washed with 0.1% bovine serum albumin (BSA) in phosphate-buffered saline (PBS) and treated with primary mAbs for 30 min at 4 °C. Thereafter, the cells were treated with Alexa Fluor 488-conjugated anti-mouse IgG (1:2000; Cell Signaling Technology, Inc., Danvers, MA, USA) or Oregon Green anti-rat IgG (1:2000; Thermo Fisher Scientific Inc.). Then, fluorescence data were collected using the SA3800 Cell Analyzer (Sony Corp., Tokyo, Japan).

### 2.5 Western blot analysis

Cell lysates (10 μg) were boiled in sodium dodecyl sulfate (SDS) sample buffer (Nacalai Tesque, Inc.). Proteins were then electrophoresed on 5%–20% polyacrylamide gels (FUJIFILM Wako Pure Chemical Corporation) and transferred onto polyvinylidene difluoride (PVDF) membranes (Merck KGaA, Darmstadt, Germany). After blocking with 4% skim milk (Nacalai Tesque, Inc.), membranes were incubated with 1 μg/mL of PMab-247, 1 μg/mL of PMab-44 (*anti*-BAP tag), and 1 μg/mL of *anti*-β-actin (clone AC-15; Sigma-Aldrich Corp., St. Louis, MO), followed by incubation with peroxidase-conjugated anti-mouse IgG (Agilent Technologies Inc., Santa Clara, CA; diluted 1:1000), and were finally developed using ImmunoStar LD (FUJIFILM Wako Pure Chemical Corporation) using a Sayaca-Imager (DRC Co. Ltd., Tokyo, Japan).

### 2.6 Immunohistochemical analyses

Cell blocks were produced using iPGell (Genostaff Co., Ltd., Tokyo, Japan). Histologic sections of 4-μm thickness were deparaffinized in xylene, then rehydrated, and autoclaved in citrate buffer (pH 6.0; Nichirei Biosciences, Inc., Tokyo, Japan) for 20 min. Then, sections were blocked using the SuperBlock T20 (PBS) Blocking Buffer (Thermo Fisher Scientific Inc.), incubated with PMab-247 for 1 h at the room temperature, and treated with the Envision + Kit for mouse (Agilent Technologies Inc.) for 30 min. Color was developed using 3,3′-diaminobenzidine tetrahydrochloride (DAB; Agilent Technologies Inc.) for 2 min, and counterstaining was performed using hematoxylin (FUJIFILM Wako Pure Chemical Corporation).

## 3 Results

**Fig. 1:**
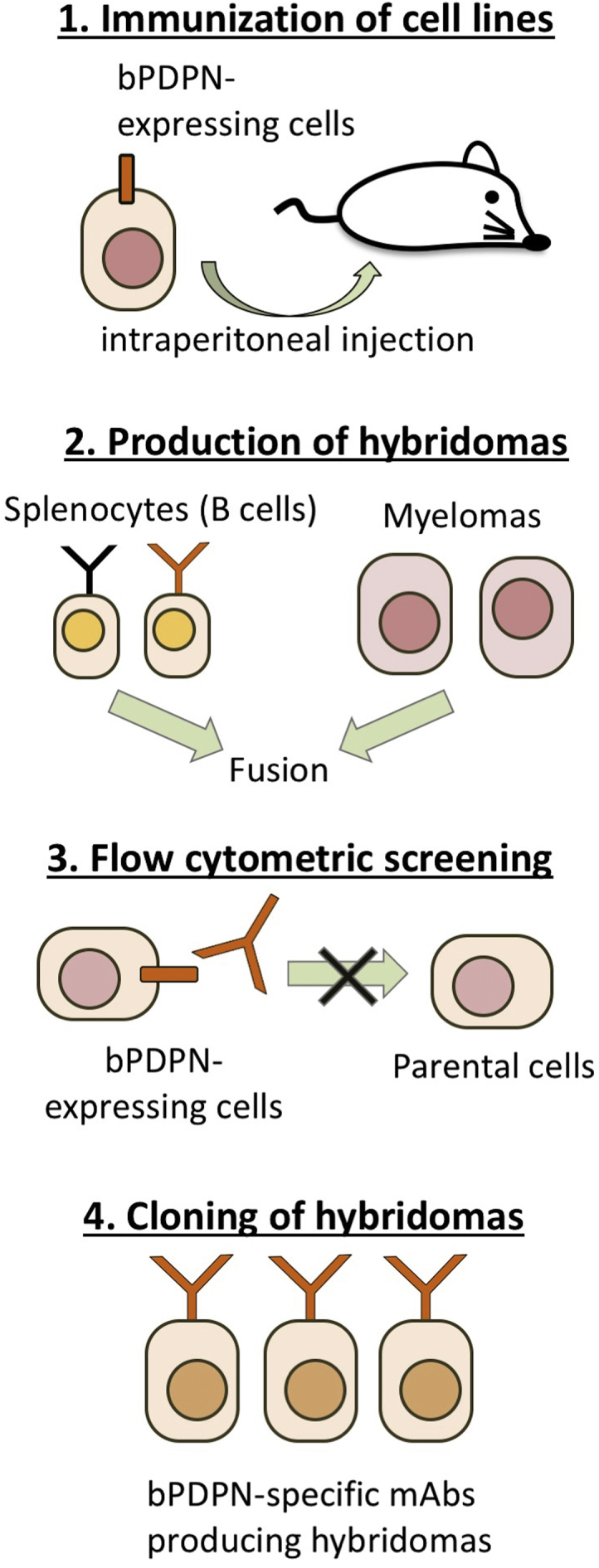
**Schematic illustration of the Cell-Based Immunization and Screening (CBIS) method.** Stable transfectants expressing the protein of interest were used as an immunogen with no purification procedure. The selection of hybridomas secreting specific mAbs was performed by flow cytometry using parental and transfectant cells.

Two mice were immunized with CHO/bPDPN cells (Fig. 1). The procedure in this study included four additional immunizations of CHO/bPDPN cells together with the Imject Alum (one mouse) or chitosan (one mouse) as adjuvants, followed by a final booster injection of CHO/bPDPN cells together with the Imject Alum (one mouse) or chitosan (one mouse). The developed hybridomas were seeded into 96-well plates and cultivated for 9 days. Wells positive for CHO/bPDPN and negative for CHO-K1 were selected by flow cytometry. The first screening approach identified strong signals from CHO/bPDPN cells and weak or no signals from CHO-K1 cells in 24 of the 480 wells (5.0%) for Imject Alum (one mouse) or in 4 of the 480 wells (0.83%) for chitosan (one mouse), indicating that Imject Alum was found to be a better adjuvant for this immunization. After several additional screenings, PMab-247 (IgG_1_, kappa) was finally selected from a mouse using chitosan.

**Fig. 2:**
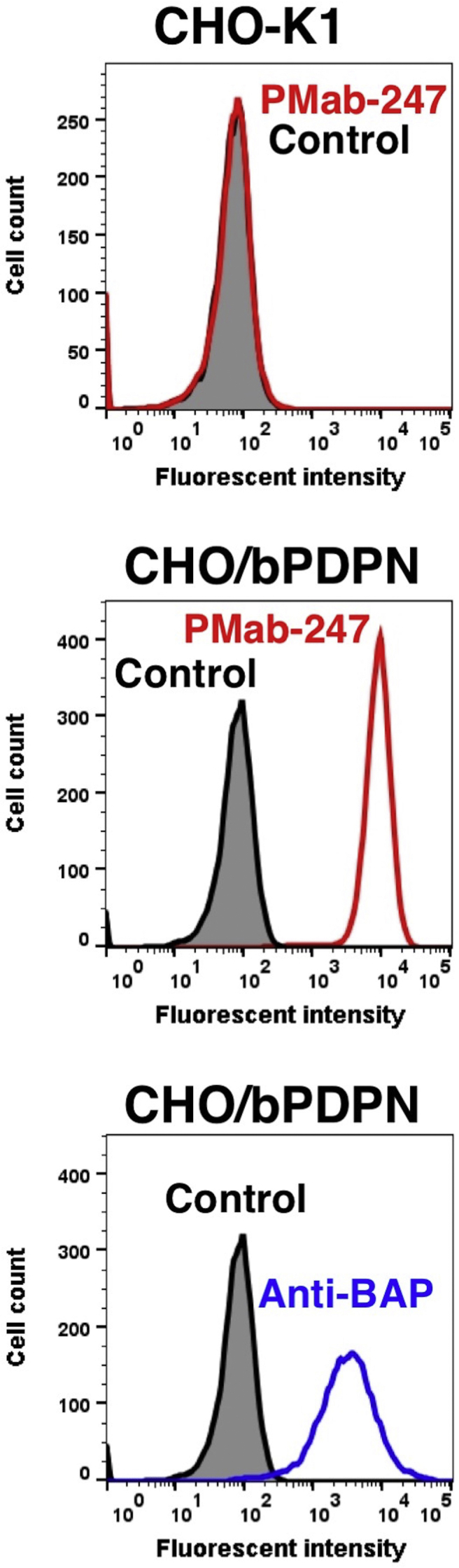
**Detection of bPDPN by flow cytometry using PMab-247.** CHO/bPDPN and CHO-K1 were treated with PMab-247 (red line) or *anti*-BAP tag (PMab-44; blue line) at a concentration of 1 μg/mL or 0.1% BSA in PBS (gray) for 30 min, followed by incubation with secondary antibodies.

**Fig. 3:**
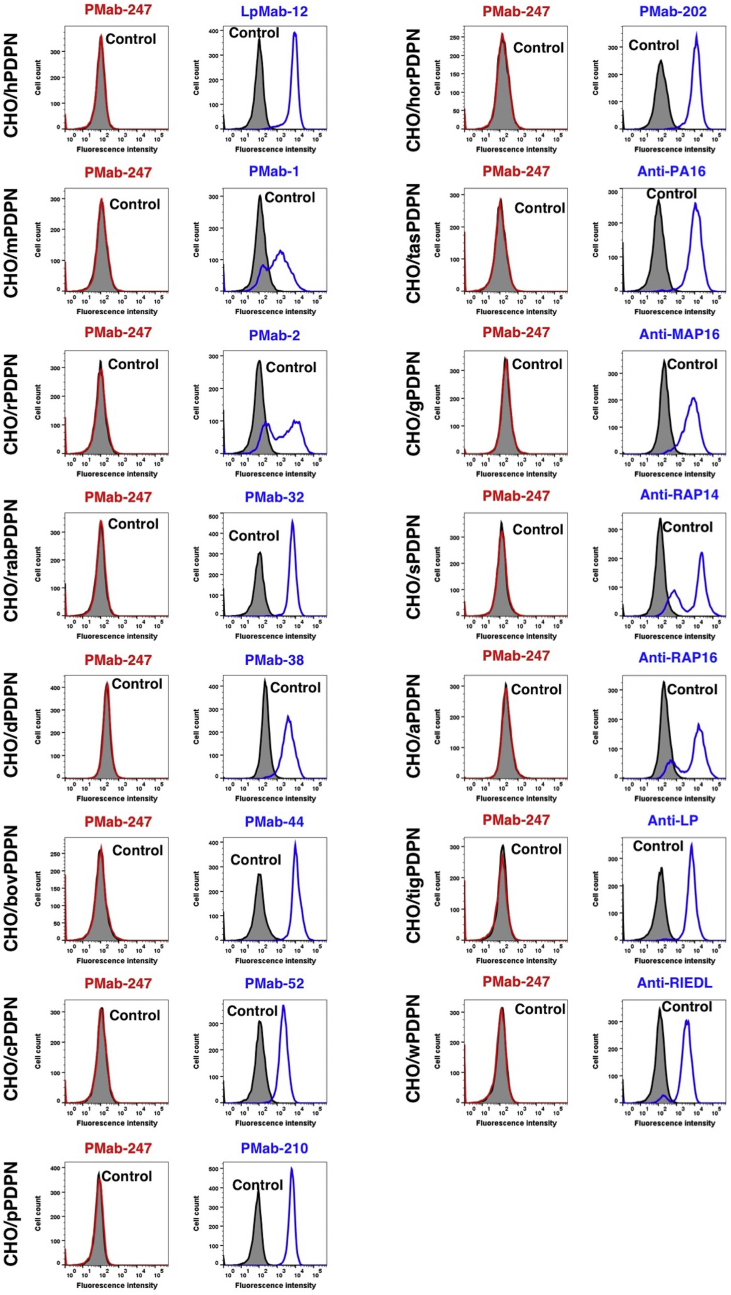
**Cross-reaction of PMab-247 to PDPNs of the other species by flow cytometry.** CHO-K1 cells transfected with PDPNs of the other species were treated with PMab-247 (red line) or each positive control (blue line) at a concentration of 1 μg/mL or 0.1% BSA in PBS (gray) for 30 min, followed by incubation with secondary antibodies.

PMab-247 recognized CHO/bPDPN cells, but showed no reaction with CHO-K1 cells, as assessed by flow cytometry (Fig. 2). PMab-247 did not cross-react with the other PDPNs (Fig. 3). The expression levels of PDPNs were confirmed by each positive control mAb.

**Fig. 4:**
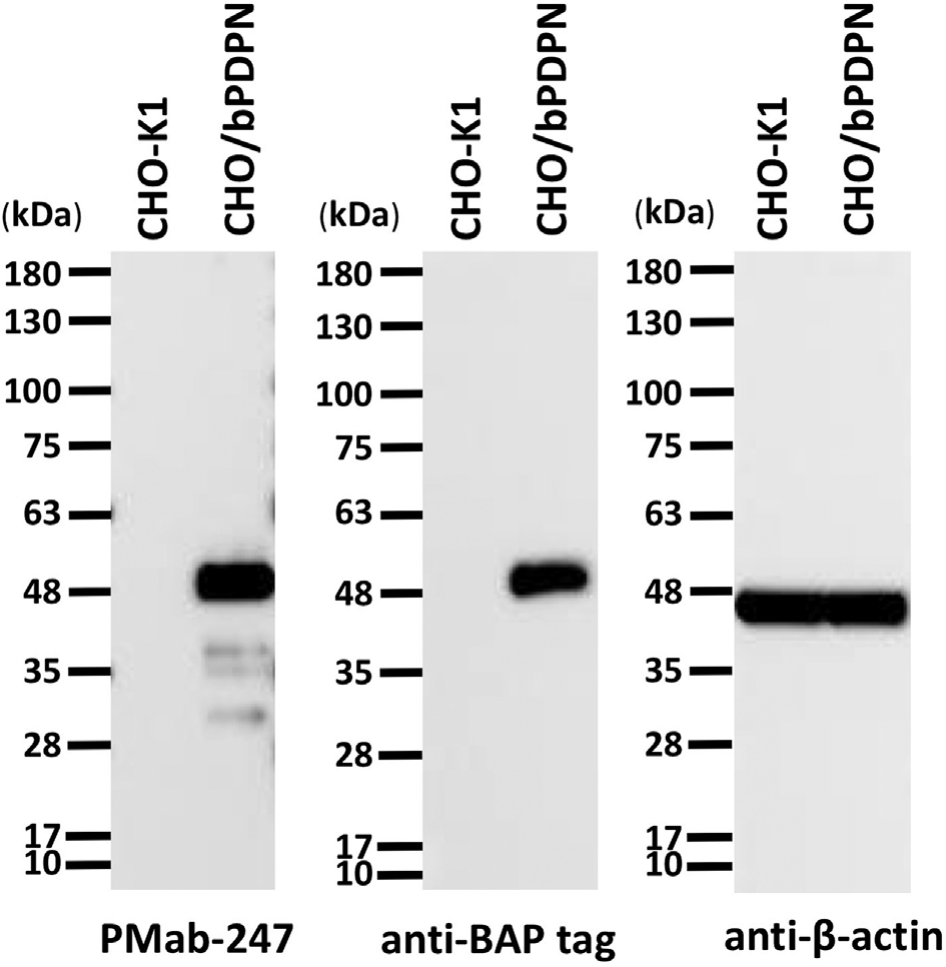
**Western blot analysis.** Cell lysates of CHO-K1 and CHO/bPDPN (10 μg) were electrophoresed and transferred onto PVDF membranes. The membranes were incubated with l μg/mL of PMab-247, 1 μg/mL of *anti*-BAP tag (PMab-44), or 1 μg/mL of anti- *β*-actin and subsequently with peroxidase-conjugated anti-mouse IgG.

**Fig. 5:**
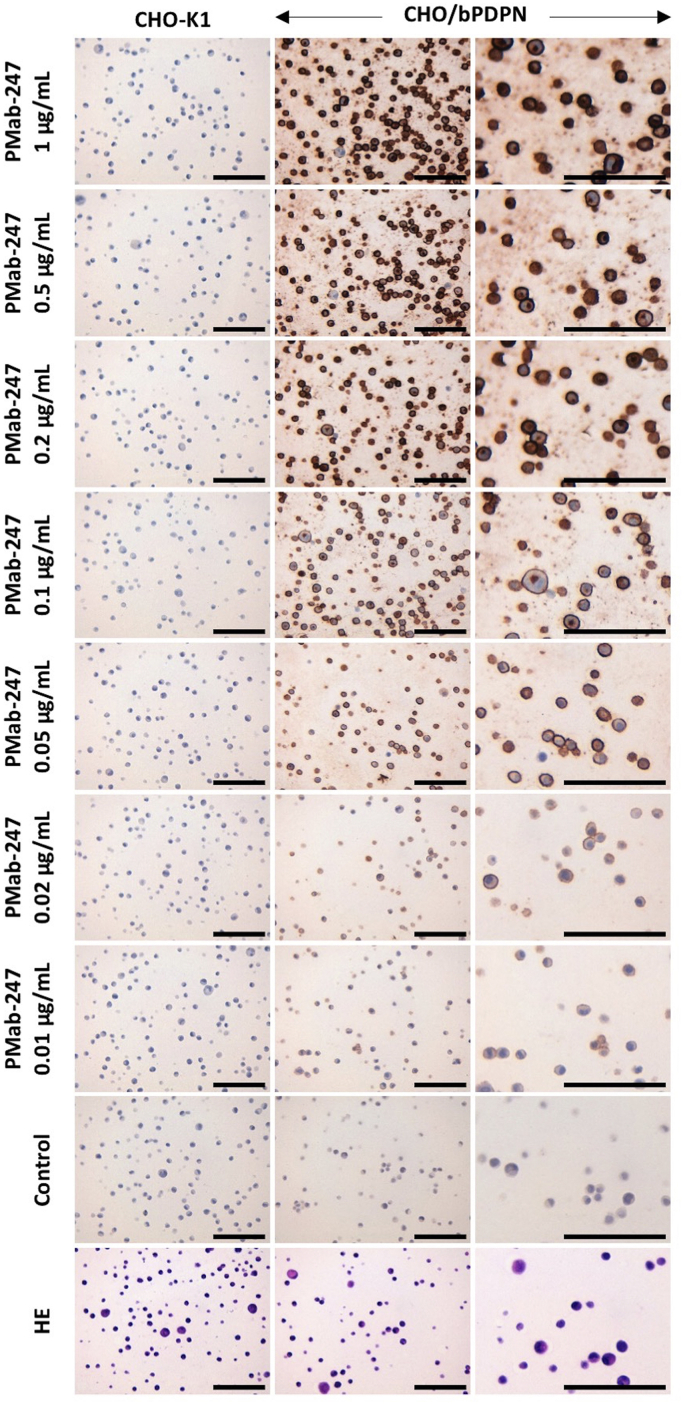
**Immunohistochemical analyses for cell lines.** CHO-K1 cells and CHO/bPDPN cells were incubated with 0.01–1 μg/mL of PMab-247 or blocking buffer, followed by the Envision + Kit. HE, Hematoxylin and eosin staining. Scale bar = 100 μm.

Western blot analysis performed using PMab-247 demonstrated that PMab-247 detects bPDPN as a 48-kDa band in CHO/bPDPN cells (Fig. 4). An *anti*-BAP tag mAb (PMab-44) also detected a 48-kDa band in CHO/bPDPN cells. The immunohistochemical analyses revealed that PMab-247 strongly stained CHO/bPDPN cells even in a concentration of 0.05 μg/mL (Fig. 5), whereas it did not react with CHO-K1 cells.

## 4 Discussion

In this study, we established PMab-247 against bPDPN, which is suitable for flow cytometry, Western blot, and immunohistochemical analyses using CBIS method. Previously, we tried to develop *anti*-bPDPN mAbs using CBIS method, and added Imject Alum as an adjuvant only in a first injection in the same way with our previous studies [14,[24], [25], [26]]. However, we could not establish *anti*-bPDPN mAbs, which are useful for immunohistochemical analyses (data not shown). Herein, we used Imject Alum or chitosan as adjuvants for every immunization, and successfully obtained high positive rates in flow cytometry, indicating that repeated use of adjuvants is also advantageous in CBIS method. The epitope of PMab-247 needs further investigation to clarify the sensitivity and specificity of PMab-247 against bPDPN.

We believe that PMab-247 should prove to be useful in elucidating the pathophysiological functions of bPDPN in some bear tumors such as osteosarcomas [27,28] or squamous cell carcinoma [29].

## Conflicts of interest

Y.K. received research funding from ZENOAQ RESOURCE CO., LTD. The other authors have no conflict of interest.
